# Incident diabetes mellitus may explain the association between sleep duration and incident coronary heart disease

**DOI:** 10.1007/s00125-017-4464-3

**Published:** 2017-11-04

**Authors:** Akiko Kishi Svensson, Thomas Svensson, Mariusz Kitlinski, Peter Almgren, Gunnar Engström, Peter M. Nilsson, Olle Melander

**Affiliations:** 1 Department of Clinical Sciences, Lund University, Skåne University Hospital, CRC, Jan Waldenströms gata 35, 20502 Malmö, Sweden; 2 Department of Diabetes and Metabolic Diseases, University of Tokyo, Tokyo, Japan; 3 Clinical Research Support Center, University of Tokyo Hospital, Tokyo, Japan; 4 Center of Innovation, University of Tokyo, Tokyo, Japan; 5 Department of Neuropsychiatry, Keio University School of Medicine, Tokyo, Japan; 6 Department of Cardiology, Skåne University Hospital, Malmö, Sweden; 7 Department of Internal Medicine, Skåne University Hospital, Malmö, Sweden

**Keywords:** Cohort, Coronary heart disease, Diabetes mellitus, Epidemiology, Incidence, Sleep duration

## Abstract

**Aims/hypothesis:**

Sleep duration is a risk factor for incident diabetes mellitus and CHD. The primary aim of the present study was to investigate, in sex-specific analyses, the role of incident diabetes as the possible biological mechanism for the reported association between short/long sleep duration and incident CHD. Considering that diabetes is a major risk factor for CHD, we hypothesised that any association with sleep duration would not hold for cases of incident CHD occurring before incident diabetes (‘non-diabetes CHD’) but would hold true for cases of incident CHD following incident diabetes (‘diabetes-CHD’).

**Methods:**

A total of 6966 men and 9378 women aged 45–73 years from the Malmö Diet Cancer Study, a population-based, prospective cohort, who had answered questions on habitual sleep duration and did not have a history of prevalent diabetes or CHD were included in the analyses. Incident cases of diabetes and CHD were identified using national registers. Sex-specific Cox proportional hazards regression models were stratified by BMI and adjusted for known covariates of diabetes and CHD.

**Results:**

Mean follow-up times for incident diabetes (*n* = 1137/1016 [men/women]), incident CHD (*n* = 1170/578), non-diabetes CHD (*n* = 1016/501) and diabetes-CHD (*n* = 154/77) were 14.2–15.2 years for men, and 15.8–16.5 years for women. In men, short sleep duration (< 6 h) was associated with incident diabetes (HR 1.35, 95% CI 1.01, 1.80), CHD (HR 1.41, 95% CI 1.06, 1.89) and diabetes-CHD (HR 2.34, 95% CI 1.20, 4.55). Short sleep duration was not associated with incident non-diabetes CHD (HR 1.35, 95% CI 0.98, 1.87). Long sleep duration (≥ 9 h) was associated with incident diabetes (HR 1.37, 95% CI 1.03, 1.83), CHD (HR 1.33, 95% CI 1.01, 1.75) and diabetes-CHD (HR 2.10, 95% CI 1.11, 4.00). Long sleep duration was not associated with incident non-diabetes CHD (HR 1.33, 95% CI 0.98, 1.80). In women, short sleep duration was associated with incident diabetes (HR 1.53, 95% CI 1.16, 2.01), CHD (HR 1.46, 95% CI 1.03, 2.07) and diabetes-CHD (HR 2.88, 95% CI 1.37, 6.08). Short sleep duration was not associated with incident non-diabetes CHD (HR 1.29, 95% CI 0.86, 1.93).

**Conclusions/interpretation:**

The associations between sleep duration and incident CHD directly reflect the associations between sleep duration and incident diabetes. Incident diabetes may thus be the explanatory mechanism for the association between short and long sleep duration and incident CHD.

**Electronic supplementary material:**

The online version of this article (10.1007/s00125-017-4464-3) contains peer-reviewed but unedited supplementary material, which is available to authorised users.

## Introduction

A suggested novel risk factor for incident diabetes mellitus is sleep duration; insufficient or excessive amounts of sleep may contribute to the development of diabetes, with prospective studies reporting that short but not long sleep duration [1–4], or both short and long sleep duration, are associated with incident diabetes [5–7]. Meta-analyses support a U-shaped association between sleep duration and incident type 2 diabetes [8, 9]. Furthermore, sleep duration predicts cardiovascular outcomes [10], and both short [11–14] and long [14, 15] sleep time is associated with incident CHD in prospective studies. The importance of sleep for cardiovascular health has prompted the American Heart Association to recently release a scientific statement outlining clinical recommendations and future research directions on sleep behaviour [16].

Any positive association between excessive or insufficient sleep duration and incident CHD must, however, be considered in the context of diabetes as a major risk factor for CHD [17]. Cardiometabolic risk factors, including prevalent type 2 diabetes, have been discussed as possible mediators for the association between sleep duration and CHD [11, 14], with prospective studies adjusting for prevalent baseline cases of diabetes in their analyses [11–15]. This may be insufficient as the same studies do not take into account the incident cases of diabetes occurring during follow-up. Incident diabetes may represent a change in the risk of developing future CHD, yet no study to date has been able to conclusively demonstrate or refute whether only those who are diagnosed with incident diabetes during follow-up constitute a specific group in which short and long sleep duration are associated with incident CHD.

Sex-stratified analyses are already an established method in cardiovascular research. However, there is emerging evidence of the importance of sex-stratification in research involving diabetes-related risk factors [18, 19] and outcomes [17, 20]. Sex-stratified analyses are thus called for to identify sex-specific diabetes and CHD risk factors and their mechanisms [17]. Sleep duration may be one such important sex-specific lifestyle-related risk factor [21].

The primary objective of the present study was thus to investigate, in sex-specific analyses, the role of incident diabetes as the possible biological mechanism for the reported association between short/long sleep duration and incident CHD. Such a crucial role of incident diabetes can be confirmed in four steps: (1) if an association between sleep duration and incident diabetes is established, and (2) an association between sleep duration and incident CHD is confirmed, it is possible to investigate whether the latter association persists when considering (3) only cases of incident CHD occurring prior to incident diabetes, and (4) only cases of incident CHD occurring after incident diabetes, respectively.

We hypothesised that any association between sleep duration and incident CHD would be significant only in individuals who, during follow-up, were diagnosed with diabetes before CHD. In accordance with this hypothesis, we expected no association between sleep duration and incident CHD occurring before incident diabetes, but an increased risk of incident CHD occurring after incident diabetes. In order to address the possibility of sex-specific risks, all analyses were conducted separately for men and women.

## Methods

The Malmö Diet and Cancer (MDC) Study is a population-based, prospective study run in the city of Malmö, Sweden, whose details have been described elsewhere [22]. In brief, men and women between the ages of 45 and 73 years were randomly selected and recruited for a baseline examination between the years 1991 and 1996. Participants’ anthropometric data and blood samples were gathered, in addition to responses to a detailed questionnaire on heredity, socioeconomic variables, social network, occupation, physical activity, alcohol consumption, smoking, diseases and medication.

At baseline, 30,447 individuals were identified in the study population (see electronic supplementary material [ESM] Fig. 1). Participants of the present study were excluded if they had a history of prevalent diabetes (*n* = 1340) or CHD (*n* = 670), provided incomplete information on sleep duration (*n* = 12,048) or provided a sleep duration that represented an outlier value of more than three interquartile ranges below or above the first and fourth quartiles respectively (*n* = 45). A total of 6966 men and 9378 women were included in the analyses. Participants were followed from the starting point until 31 December 2010, with person-years calculated from the starting point to the date of the incident event, loss to follow-up or the end of the follow-up period, whichever came first.

The MDC study was approved by the ethics committee at Lund University, and all participants provided written informed consent.

### Sleep duration

Habitual sleep duration was assessed through two open questions asking participants how long they slept on weekdays and weekends, respectively. The two questions were formulated as: (a) ‘How many hours do you usually sleep per night during a typical week (Monday–Friday)?’ and (b) ‘How many hours do you usually sleep per night during a typical weekend (Saturday–Sunday)?’ A weighted average sleep duration [(weekday × 5) + (weekend × 2)/7] was calculated for individuals who responded to both questions (*n* = 16,344), thereby allowing the subsequent construction of a categorical variable consisting of five sleep duration groups (< 6 h, 6–7 h, 7–8 h, 8–9 h and ≥ 9 h). Because only 0.9% (*n* = 146) of the population slept for < 5 h, short and long sleep duration were defined as < 6 h and ≥ 9 h, respectively. The reference category (7–8 h) was chosen on the basis of the lowest incidence rates of all endpoints in this particular group.

### Incident diabetes mellitus

Incident diabetes was defined as new-onset diabetes in individuals without prevalent diabetes at baseline. Prevalent diabetes at the MDC baseline examination was defined as any of the following: having a measured fasting blood glucose ≥ 6.1 mmol/l (corresponding to fasting plasma glucose concentration ≥ 7 mmol/l) at the MDC baseline examination, or a self-reported history of physician-diagnosed diabetes, or use of diabetes medication according to the MDC baseline questionnaire, or being diagnosed and registered in any of the local or national diabetes registries as previously described [23].

The process of endpoint retrieval has been described in detail elsewhere [24]. In brief, individuals with incident diabetes were identified through linkage of a 10-digit national personal identification number with six local and national registers: the Malmö HbA_1c_ register, the Regional Diabetes 2000 register of the Scania region [25], the Swedish National Diabetes Register [26], the Swedish National Inpatient Register [27], the Swedish Cause of Death Register [28] and the Swedish Prescribed Drug Register [29]. Apart from being captured in these registers, individuals with incident diabetes could also be captured by having a fasting plasma glucose concentration ≥ 7 mmol/l or a 120 min plasma glucose value of > 11.0 mmol/l in subpopulations of the MDC participating in a MDC re-examination [30] or the Malmö Preventive Project re-examination. [31]

### Incident coronary heart disease

Time to first occurrence of a CHD event was defined as a first fatal or non-fatal myocardial infarction, coronary artery bypass graft (CABG) or percutaneous coronary intervention (PCI). All events were identified through linkage of a 10-digit national personal identification number with three registries validated for classification of outcomes as described elsewhere [32, 33]: the Swedish National Discharge Registry, the Swedish Cause of Death Register, and the Swedish Coronary Angiography and Angioplasty Registry. CABG and PCI were classified using the national classification of surgical procedures operation codes (KKÅ or Op6): 3065, 3066, 3068, 3080, 3092, 3105, 3127, 3158 for CABG, and FNG02 and FNG05 for PCI. A coronary event was defined according to the ICD-9 (www.icd9data.com/2007/Volume1) and ICD-10 (www.who.int/classifications/icd/en/) revisions with fatal or non-fatal myocardial infarction or death due to CHD corresponding to codes 410, 412, and 414 (ICD-9), and I21-I23 and, I25 (ICD-10).

### Incident CHD in relation to incident diabetes

In order to elucidate the role of diabetes as the explanatory mechanism for the association between sleep duration and CHD, two additional endpoint variables were created: (1) ‘non-diabetes CHD’, defined as an incident CHD event occurring in participants without incident diabetes, or in those in whom incident diabetes occurred after incident CHD; and (2) ‘diabetes-CHD’, defined as incident CHD diagnosed on the same day, or following a diagnosis of incident diabetes.

### Statistical analyses

Sex-specific risks for the association between sleep duration and incident diabetes were estimated using Cox proportional hazards regression. The minimally adjusted models were adjusted for age (continuous) and socioeconomic index at starting point. Multivariable models were additionally adjusted for marital status (married, single, divorced or widowed), smoking (never, past and current smoker: < 20 cigarettes/day, or ≥ 20 cigarettes/day), alcohol intake (none, 0.1–1.6, 1.7–7.2, 7.3–15.4 or ≥ 15.5 g ethanol/day), physical activity, hypertension, use of lipid-lowering medication, shift work (yes/no), psychological stress (low, intermediate and high) and sleep quality. Missing data were addressed through the construction of dummy variables. All multivariable models were stratified by four categories of BMI (< 18.5 kg/m^2^, 18.5–24.9 kg/m^2^, 25–29.9 kg/m^2^ and ≥ 30 kg/m^2^), using the Stata option ‘strata()’ to account for different baseline hazards across BMI categories.

Socioeconomic index was categorised according to the Swedish socioeconomic classification [34] (manual worker, low and intermediate level non-manual worker, higher level non-manual worker, other [self-employed including farmers] and unemployed). Physical activity was defined as leisure time physical activity based on 18 items adapted from the Minnesota Leisure Time Physical Activity instrument and has been described in detail elsewhere [35]. Hypertension was defined as systolic blood pressure ≥ 140 mmHg, diastolic blood pressure ≥ 90 mmHg or the use of antihypertensive medication (angiotensin-converting enzyme inhibitors, β-blockers, calcium antagonists or diuretics). Psychological stress was constructed from validated questions assessing psychosocial work characteristics measuring job strain, and from one question assessing non-occupational stress. Sleep quality was determined through four questions assessing (1) difficulty initiating sleep, (2) difficulty maintaining sleep, (3) early morning awakening, and (4) not feeling rested.

Sensitivity analyses excluded the first 3 years of follow-up to minimise the chance of reverse causation.

All statistical analyses were performed using Stata version 13.1 SE (StataCorp LP, College Station, TX, USA). *p* values were two-tailed and considered significant if *p* < 0.05.

## Results

Compared with those who did not provide information on sleep duration, the group that was included in all analyses had a higher proportion of men (42.6% vs 32.4%). Moreover, compared with those who were included (mean age and SD for both men and women, 57.3 ± 6.0 years), men who did not provide information on sleep duration were older (61.3 ± 7.8 years), whereas women were slightly younger (57.0 ± 9.6 years).

Mean sleep duration for men and women was 7.3 h (SD ± 0.9 h). Table 1 summarises baseline characteristics stratified according to sex. The largest proportion of men (42.9%) and women (40.9%) had a sleep duration of 7–8 h. Compared with those with short (< 6 h) and long (≥ 9 h) sleep durations, men and women in the reference category (7–8 h) were less likely to be obese, do high levels of physical activity or shift work, and smoke more than 20 cigarettes/day, and more likely to be married and report low levels of psychological stress. Men who reported 7–8 h of sleep were also less likely to be hypertensive and less likely to use lipid-lowering medication.

**Table 1:** Baseline characteristics for men and women according to sleep duration

Variable	Men (*n* = 6966)						Women (*n* = 9378)					
	Sleep duration (h)					*p* value	Sleep duration (h)					*p* value
	< 6	6–7	7–8	8–9	≥ 9		< 6	6–7	7–8	8–9	≥ 9	
Number of individuals	305	1484	2988	1897	292		460	1819	3835	2843	421	
Proportion of population (%)	4.4	21.3	42.9	27.2	4.2		4.9	19.4	40.9	30.3	4.5	
Age (mean years ± SD)	57.0 ± 5.9	56.6 ± 5.7	56.7 ± 5.9	58.4 ± 6.1	59.7 ± 5.9	< 0.001	58.3 ± 6.0	57.2 ± 6.0	56.6 ± 5.9	57.9 ± 6.1	58.4 ± 6.2	< 0.001
Socioeconomic index (%)						< 0.001						< 0.001
Manual worker	43.9	33.6	29.5	33.6	37.0		43.0	39.3	33.7	37.9	42.8	
Lower and intermediate non-manual worker	21.6	33.4	35.7	31.9	28.1		39.4	45.0	48.5	44.0	33.5	
Higher non-manual worker	10.2	11.0	13.4	9.1	6.5		4.8	5.8	7.0	5.3	4.3	
Other (self-employed and farmers)	17.4	15.9	15.9	18.5	21.9		7.0	5.2	6.2	6.7	9.5	
Unemployed	5.6	5.8	5.4	6.7	6.2		3.5	3.6	3.8	4.9	7.1	
Missing information	1.3	0.4	0.2	0.3	0.3		2.4	1.2	0.8	1.2	2.9	
Smoking status (%)						0.02						< 0.001
Never	29.2	27.9	30.9	28.5	20.9		41.7	42.3	46.2	47.9	40.9	
Past	37.1	40.4	40.6	42.2	40.8		30.0	26.2	26.1	26.0	27.6	
Current < 20 cigarettes/day	11.2	11.1	10.8	10.5	14.0		13.7	18.1	17.0	15.2	18.5	
Current ≥ 20 cigarettes/day	22.6	20.6	17.7	18.8	24.3		14.6	13.5	10.7	10.8	13.1	
Missing information	0.0	0.1	0.0	0.1	0.0		0.0	0.0	0.0	0.1	0.0	
Alcohol consumption (%)						< 0.001						< 0.001
None	18.0	11.5	9.4	12.3	17.5		24.8	23.0	15.8	18.0	23.8	
0.1–1.6 g ethanol day	6.2	6.7	5.1	6.1	6.2		13.0	12.9	11.5	12.8	13.8	
1.7–7.2 g ethanol/day	13.4	16.9	17.8	19.1	19.5		26.5	25.8	30.1	29.2	30.6	
7.3–15.4 g ethanol/day	18.7	22.6	26.6	24.1	21.6		22.4	23.1	27.6	25.6	16.6	
≥ 15.5 g ethanol/day	40.0	41.2	40.0	37.1	33.2		11.3	13.9	14.4	13.4	14.5	
Missing information	3.6	1.2	1.2	1.3	2.1		2.0	1.3	0.7	1.0	0.7	
BMI (%)						< 0.001						0.03
< 18.5 kg/m^2^	1.3	0.9	0.3	0.7	1.0		2.2	1.3	1.3	1.8	1.7	
18.5–24.9 kg/m^2^	34.4	39.0	41.0	37.0	37.0		49.1	51.8	54.2	52.3	48.9	
25.0–29.9 kg/m^2^	47.5	47.0	49.1	50.1	45.9		31.3	33.4	33.0	32.8	33.7	
≥ 30.0 kg/m^2^	16.4	12.8	9.5	12.2	16.1		17.4	13.5	11.4	13.0	15.7	
Missing information	0.3	0.3	0.1	0.1	0.0		0.0	0.1	0.1	0.2	0.0	
Physical activity (%)						0.002						< 0.001
Quartile 1 (low physical activity)	32.5	23.9	25.2	25.1	27.4		24.6	26.3	23.1	24.0	31.1	
Quartile 2	21.3	24.3	25.8	23.3	20.9		24.1	24.9	27.6	25.3	22.3	
Quartile 3	18.4	23.7	23.7	23.9	18.5		24.6	23.1	26.2	25.3	19.5	
Quartile 4 (high physical activity)	26.2	27.0	24.7	27.1	31.9		25.0	24.8	22.4	24.4	25.7	
Missing information	1.6	1.2	0.5	0.7	1.4		1.7	0.9	0.7	1.0	1.4	
Hypertension (%)	44.6	45.6	43.5	45.8	55.1	0.004	42.0	36.0	36.1	36.9	40.1	NS
Lipid-lowering medication (%)	2.6	2.1	2.1	2.8	4.8	0.04	3.0	1.4	1.5	1.4	1.9	NS
Shift work (%)	38.0	33.7	28.7	32.0	45.6	< 0.001	33.0	28.9	23.7	27.6	33.5	< 0.001
Missing information	3.3	0.8	0.6	0.3	1.4		3.5	2.1	1.5	2.4	4.0	
Marital status (%)						0.002						< 0.001
Married	64.9	71.2	74.4	74.5	65.4		55.4	59.3	64.5	64.9	61.3	
Single	12.1	10.4	10.1	10.4	12.3		7.6	9.4	7.9	7.7	8.8	
Divorced	18.4	16.0	13.6	12.4	18.5		24.1	19.5	19.0	19.4	21.1	
Widowed	4.6	2.4	2.0	2.6	3.8		12.8	11.8	8.6	7.8	8.6	
Missing information	0.0	0.0	0.0	0.0	0.0		0.0	0.1	0.1	0.1	0.2	
Psychological stress (%)						< 0.001						< 0.001
Low	29.2	35.2	40.8	37.9	33.2		18.0	25.0	30.5	30.2	21.4	
Intermediate	32.1	36.9	32.8	34.7	34.3		41.7	37.7	35.3	34.9	35.9	
High	21.0	12.7	8.2	9.8	11.3		21.7	18.1	13.8	11.7	15.2	
Missing information	17.7	15.2	18.2	17.7	21.2		18.5	19.2	20.4	23.2	27.6	
Sleep quality (%)						< 0.001						< 0.001
Good	50.8	74.4	86.1	87.8	83.2		29.1	55.9	76.6	79.5	77.7	
Poor	48.2	25.1	13.2	11.4	15.4		70.4	43.3	22.7	19.5	20.7	
Missing information	1.0	0.5	0.7	0.7	1.4		0.4	0.8	0.7	1.0	1.7	

χ^2^ test was used for categorical variables and ANOVA for continuous variables

NS, non-significant

### Incident diabetes

Mean follow-up time for analysis of incident diabetes was 15.0 years for men and 16.0 years for women. During follow-up, 16.3% of men (*n* = 1137) and 10.8% of women (*n* = 1016) developed diabetes.

In the multivariable analysis for men, short sleep duration was borderline significantly associated with incident diabetes (HR 1.26, 95% CI 0.96, 1.65), while long sleep duration was significantly associated with an increased risk of incident diabetes (HR 1.31, 95% CI 1.00, 1.71) compared with the reference category (Table 2). When excluding the first 3 years of follow-up, men with short (HR 1.35, 95% CI 1.01, 1.80) and long (HR 1.37, 95% CI 1.03, 1.83) sleep durations had an increased risk of incident diabetes.

**Table 2:** HRs and CIs for incident diabetes mellitus according to sleep duration for men and women

	Men (*n* = 6966)					Women (*n* = 9378)				
Incident diabetes	Sleep duration (h)					Sleep duration (h)				
	< 6	6–7	7–8	8–9	≥ 9	< 6	6–7	7–8	8–9	≥ 9
Person-years	4310	21,925	45,819	28,153	4004	7018	29,036	62,268	45,558	6511
No. (events)	305 (65)	1484 (250)	2988 (459)	1897 (299)	292 (64)	460 (77)	1819 (197)	3835 (370)	2843 (321)	421 (51)
Model 1, HR (95% CI)	1.47 (1.14, 1.91)**	1.14 (0.97, 1.33)	Reference	1.03 (0.89, 1.19)	1.53 (1.18, 1.99)**	1.74 (1.36, 2.22)***	1.10 (0.92, 1.31)	Reference	1.12 (0.97, 1.30)	1.19 (0.89, 1.60)
Model 2, HR (95% CI)	1.26 (0.96, 1.65)	1.05 (0.90, 1.23)	Reference	0.96 (0.83, 1.12)	1.31 (1.00, 1.71)*	1.55 (1.20, 2.01)***	1.04 (0.87, 1.24)	Reference	1.15 (0.98, 1.33)	1.15 (0.86, 1.55)
Model 3, HR (95% CI)	1.35 (1.01, 1.80)*	1.12 (0.95, 1.32)	Reference	1.00 (0.85, 1.17)	1.37 (1.03, 1.83)*	1.53 (1.16, 2.01)**	1.05 (0.87, 1.27)	Reference	1.13 (0.96, 1.32)	1.03 (0.74, 1.44)

Model 1 was adjusted for age and socioeconomic index

Model 2 was stratified by BMI category and adjusted for age, socioeconomic index, marital status, smoking, alcohol intake, physical activity, hypertension, use of lipid-lowering medication, shift work, psychological stress and sleep quality

Model 3 additionally excluded the first 3 years of follow-up

*p < 0.05, **p < 0.01, ***p < 0.001 vs reference

Among women, short sleep duration was associated with incident diabetes in the fully adjusted model (HR 1.55, 95% CI 1.20, 2.01) and when excluding the first 3 years of follow-up (HR 1.53, 95% CI 1.16, 2.01) (Table 2). Long sleep duration was not associated with incident diabetes in women.

### Incident CHD

Mean follow-up time for analysis of incident CHD was 15.2 years for men and 16.5 years for women. During follow-up, 16.8% of men (*n* = 1170) and 6.2% of women (*n* = 578) developed CHD.

In the fully adjusted models for men, short sleep duration was associated with incident CHD (HR 1.32, 95% CI 1.01, 1.73) (Table 3). When excluding the first 3 years of follow-up, both short (HR 1.41, 95% CI 1.06, 1.89) and long (HR 1.33, 95% CI 1.01, 1.75) sleep duration were associated with an increased risk of CHD.

**Table 3:** HRs and CIs for incident CHD according to sleep duration for men and women

	Men (*n* = 6966)					Women (*n* = 9378)				
Incident CHD	Sleep duration (h)					Sleep duration (h)				
	< 6	6–7	7–8	8–9	≥ 9	< 6	6–7	7–8	8–9	≥ 9
Person-years	4396	22,435	46,363	28,209	4167	7447	29,683	63,690	46,888	6709
No. (events)	305 (65)	1484 (247)	2988 (445)	1897 (346)	292 (67)	460 (43)	1819 (117)	3835 (205)	2843 (177)	421 (36)
Model 1, HR (95% CI)	1.47 (1.13, 1.90)**	1.16 (1.00, 1.36)	Reference	1.14 (0.99, 1.31)	1.38 (1.06, 1.79)*	1.53 (1.10, 2.13)*	1.14 (0.91, 1.44)	Reference	1.02 (0.83, 1.25)	1.37 (0.96, 1.95)
Model 2, HR (95% CI)	1.32 (1.01, 1.73)*	1.10 (0.94, 1.29)	Reference	1.09 (0.95, 1.26)	1.15 (0.88, 1.49)	1.41 (1.00, 1.99)*	1.07 (0.85, 1.35)	Reference	1.02 (0.83, 1.25)	1.31 (0.91, 1.87)
Model 3, HR (95% CI)	1.41 (1.06, 1.89)*	1.12 (0.94, 1.33)	Reference	1.15 (0.99, 1.34)	1.33 (1.01, 1.75)*	1.46 (1.03, 2.07)*	1.06 (0.83, 1.34)	Reference	0.96 (0.78, 1.19)	1.32 (0.91, 1.91)

Model 1 was adjusted for age and socioeconomic index

Model 2 was stratified by BMI category and adjusted for age, socioeconomic index, marital status, smoking, alcohol intake, physical activity, hypertension, use of lipid-lowering medication, shift work, psychological stress and sleep quality

Model 3 additionally excluded the first 3 years of follow-up

*p < 0.05, **p < 0.01 vs reference

For women, short sleep duration was associated with an increased risk of CHD in the fully adjusted model (HR 1.41, 95% CI 1.00, 1.99), and in the sensitivity analysis (HR 1.46, 95% CI 1.03, 2.07) (Table 3). Long sleep duration was not associated with any increase in risk of CHD.

### Incident non-diabetes CHD

Mean follow-up time for analysis of incident non-diabetes CHD was 14.2 years for men and 15.8 years for women. During follow-up, 14.6% of men (*n* = 1016) and 5.3% of women (*n* = 501) developed non-diabetes CHD.

In the multivariable model for men, neither short (HR 1.23, 95% CI 0.91, 1.66) nor long (HR 1.13, 95% CI 0.84, 1.50) sleep duration were associated with an increased risk of incident non-diabetes CHD (ESM Table 1). When excluding the first 3 years of follow-up, the results for short (HR 1.35, 95% CI 0.98, 1.87) and long (HR 1.33, 95% CI 0.98, 1.80) sleep duration remained non-significantly associated with incident non-diabetes CHD.

In the multivariable model for women, there was no significant association between short (HR 1.24, 95% CI 0.83, 1.84) or long (HR 1.28, 95% CI 0.87, 1.88) sleep duration and incident non-diabetes CHD (ESM Table 1). When excluding the first 3 years of follow-up, the results for short (HR 1.29, 95% CI 0.86, 1.93) and long (HR 1.34, 95% CI 0.90, 1.98) sleep duration remained non-significantly associated with incident non-diabetes CHD.

### Incident diabetes-CHD

Mean follow-up time for analysis of incident diabetes-CHD was 14.4 years for men and 15.9 years for women. During follow-up, 2.2% of men (*n* = 154) and 0.8% of women (*n* = 77) developed diabetes-CHD.

In the multivariable model for men, short (HR 2.37, 95% CI 1.22, 4.60) and long (HR 2.21, 95% CI 1.16, 4.19) sleep duration was associated with an increased risk of incident diabetes-CHD (Table 4). When excluding the first 3 years of follow-up, both short (HR 2.34, 95% CI 1.20, 4.55) and long (HR 2.10, 95% CI 1.11, 4.00) sleep duration were associated with an increased risk of incident diabetes-CHD.

**Table 4:** HRs and CIs for incident CHD occurring after incident diabetes mellitus according to sleep duration for men and women

	Men (*n* = 6966)					Women (*n* = 9378)				
Incident diabetes-CHD	Sleep duration (h)					Sleep duration (h)				
	< 6	6–7	7–8	8–9	≥ 9	< 6	6–7	7–8	8–9	≥ 9
Person-years	4154	21,077	44,252	26,919	3854	6980	28,652	61,680	45,047	6422
No. (events)	305 (12)	1484 (29)	2988 (53)	1897 (48)	292 (12)	460 (12)	1819 (17)	3835 (24)	2843 (19)	421 (5)
Model 1, HR (95% CI)	2.44 (1.30, 4.58)**	1.19 (0.76, 1.88)	Reference	1.38 (0.93, 2.04)	2.37 (1.26, 4.47)**	3.90 (1.94, 7.83)***	1.42 (0.76, 2.65)	Reference	0.94 (0.51, 1.72)	1.59 (0.60, 4.20)
Model 2, HR (95% CI)	2.37 (1.22, 4.60)*	1.06 (0.67, 1.68)	Reference	1.26 (0.85, 1.88)	2.21 (1.16, 4.19)*	2.93 (1.39, 6.16)**	1.27 (0.67, 2.40)	Reference	1.02 (0.55, 1.88)	1.56 (0.58, 4.16)
Model 3, HR (95% CI)	2.34 (1.20, 4.55)*	1.06 (0.67, 1.68)	Reference	1.23 (0.82, 1.83)	2.10 (1.11, 4.00)*	2.88 (1.37, 6.08)**	1.19 (0.62, 2.28)	Reference	0.98 (0.52, 1.81)	1.22 (0.42, 3.60)

Model 1 was adjusted for age and socioeconomic index

Model 2 was stratified by BMI category and adjusted for age, socioeconomic index, marital status, smoking, alcohol intake, physical activity, hypertension, use of lipid-lowering medication, shift work, psychological stress and sleep quality

Model 3 additionally excluded the first 3 years of follow-up

*p < 0.05, **p < 0.01, ***p < 0.001 vs reference

In women, short sleep duration was associated with an increased risk of diabetes-CHD in both the multivariable model (HR 2.93, 95% CI 1.39, 6.16) and the sensitivity analysis (HR 2.88, 95% CI 1.37, 6.08) (Table 4).

## Discussion

Our study is the first of its kind to consider incident diabetes on the pathway between self-reported sleep duration and CHD risk. The associations between sleep duration and incident CHD directly reflect the associations between sleep duration and incident diabetes, and when taken together with the strong association between sleep duration and diabetes-CHD, our study convincingly demonstrates that incident diabetes is the most probable explanatory biological mechanism for the positive associations found between sleep duration and CHD.

The association between short sleep duration and incident diabetes in our study is consistent with previous prospective findings [1–7]. Although both BMI and hypertension have been mentioned as possible mediators [6] for the association between short sleep duration and incident diabetes, the association between short sleep duration and incident diabetes in our study persisted in both men and women despite BMI-stratified analyses and adjustment for a large number of known diabetes risk factors and covariates, including hypertension. Prospective studies with repeated measurements of BMI and blood pressure are, however, urged to investigate the importance of any change in these variables over time for the association between sleep duration, incident diabetes and incident CHD, respectively.

The increased risk of CHD with short sleep duration [11–14] is also consistent with previous prospective studies and could easily have been argued as an increased risk independent of diabetes considering the exclusion of individuals with prevalent diabetes from our analyses. However, we chose to take into account the association between habitually short sleep duration and incident diabetes and opted for an approach that (1) censored cases of incident CHD if these occurred after incident diabetes (non-diabetes CHD), thereby obliterating any significant association between short sleep duration and CHD; and (2) considered only cases of incident CHD that followed incident diabetes (diabetes-CHD), which in turn showed significant positive associations between short sleep duration and CHD despite the low number of cases. This novel approach recognises the important role of incident diabetes as the explanatory mechanism for any association between short sleep duration and incident CHD in both men and women.

Our study also found that long sleep duration (≥ 9 h) in men is associated with incident diabetes and diabetes-CHD, but not with non-diabetes CHD. Contrary to the situation with short sleep duration, the associations between long sleep duration and incident diabetes and CHD, respectively, have been suggested to result from comorbidity and residual confounding [5, 10] or from reverse causation bias [2, 14]. In order to address the problem of reverse causation, our study excluded individuals with prevalent diabetes and CHD, and allowed for sensitivity analyses that further excluded the first 3 years of follow-up. Despite this approach, long sleep duration in men remained significantly positively associated with incident diabetes, incident CHD and incident diabetes-CHD, but not with incident non-diabetes CHD. The main reason for a null finding between long sleep duration and incident CHD in women is most likely due to the lack of any association between long sleep duration and incident diabetes in this group, a finding consistent with one previous report [1]. We therefore propose for the first time that incident diabetes is also the responsible mechanism for the observed association between long sleep duration and incident CHD in men.

The findings are strong and point toward diabetes as the explanatory biological mechanism for the association between sleep duration and CHD, and the fact that this association may differ between men and women. The significance of this finding is threefold. First, it highlights that sleep duration should be considered an important behavioural risk factor for incident diabetes. Second, the relevance of our findings is not limited to CHD, owing to the importance of diabetes as a major risk factor for micro- and macrovascular disease. Sleep interventions in individuals who are habitually short or long sleepers thus have the potential to greatly impact health outcomes and should be considered on a par with advice on physical exercise. Indeed, the American Heart Association has recently published a scientific statement [16] outlining clinical recommendations and research priorities on sleep behaviour owing to the strong association between sleep and cardiovascular health. Third, the present results strengthen the need for sex-stratified analyses with regard to diabetes risk factors and CHD complications. The main sex difference that emerges from our study is that long sleep duration is a risk factor for incident diabetes and incident diabetes-CHD in men but not women. Such differences between men and women may be due to underlying biological as well as psychosocial influences [36], which may determine sex-specific diabetes risk factors [18]. Indeed, sex differences in the association between sleep duration and incident diabetes have already been reported [1], and the impact of sleep duration on body composition may differ according to sex [21]. Mechanistic studies are required to explain the sex-specific effects of sleep duration and their association with incident diabetes and incident CHD, respectively. Future research on this topic is thus highly warranted.

There are a few limitations to this study. First, sleep was assessed through self-reported questionnaires, with possible overestimation of sleep duration. However, any non-differential misclassification of sleep duration among our participants would consequently result in a subsequent underestimation of our study findings. Additionally, when considering the nature and size of our study, no other feasible methods of assessing sleep duration exist. Second, the lack of an association between long sleep duration and the diabetes-CHD endpoint in women could be due to a lack of statistical power because of the small number of individuals involved. Studies with a larger population and longer follow-up times are encouraged to conduct sex-stratified analyses and attempt to replicate our results. Third, the MDC study does not contain information on mental illness, and we were therefore unable to adjust for this in our analyses. Fourth, the group of individuals included in analyses differed in age and sex from those who had not provided information on sleep duration. Those included for analysis included a higher proportion of men, and they were also younger than the excluded men. Conversely, the women included were slightly older than their excluded counterparts. This may have biased our findings. Fifth, the generalisability of results to other populations may be limited. Ethnic differences in the association between sleep duration and incident diabetes have been shown in previous research [2], and different ethnicities may require different amounts of sleep before such behaviours constitute a health hazard.

Despite such limitations, this study has a number of strengths. First, incident diabetes and incident CHD were established using nationwide registers, with very high accuracy. Second, the MDC questionnaire asks about habitual sleep duration, which would be the question of choice if sleep duration were assessed by healthcare professionals. Third, we have used a population that is highly representative of the Swedish general population, and we adjusted for a large number of covariates associated with incident diabetes and CHD. Finally, we adjusted for sleep quality as poor sleep quality may confound any association between short or long sleep duration and health outcomes. With the exception of one prospective study [3], sleep quality has not been considered when positive associations have been reported between sleep duration and incident diabetes.

### Conclusion

The results of this population-based study demonstrate for the first time that the association between sleep duration and incident CHD may be explained by incident diabetes. Interventions targeting sleep duration could thus have a significant impact on health outcomes when considering the importance of diabetes for a wide range of diseases.

## Electronic supplementary material

ESM (PDF 284 kb)

## Abbreviations

CABG: Coronary artery bypass graft
MDC: Malmö Diet and Cancer
PCI: Percutaneous coronary intervention
