# Supplementary material for: Incident diabetes mellitus may explain the association between sleep duration and incident coronary heart disease
Source: Diabetologia. 2017 Nov 4;61(2):331–41. doi: 10.1007/s00125-017-4464-3 (PMC6448951; doi:10.1007/s00125-017-4464-3)
Supplement: Supplementary file 1 — (PDF 284 kb) [file 125_2017_4464_MOESM1_ESM.pdf]

ESM Table 1 Hazard ratios and confidence intervals for incident coronary heart disease occurring before incident diabetes mellitus (non-diabetes CHD) according to sleep duration for men and women respectively.

Incident non-diabetes CHD	Men (n=6966)					Women (n=9378)				
	Sleep duration (h)					Sleep duration (h)				
	<6	6-7	7-8	8-9	≥9	<6	6-7	7-8	8-9	≥9
Person-years	4053	20,827	43,710	26,510	3760	6909	28,519	61,436	44,853	6368
No. (events)	305 (53)	1484 (218)	2988 (392)	1897 (298)	292 (55)	460 (31)	1819 (100)	3835 (181)	2843 (158)	421 (31)
Model 1, HR	1.39	1.18	Reference	1.11	1.33	1.32	1.11	Reference	1.04	1.34
(95% CI)	(1.05, 1.86)*	(1.00, 1.40)*		(0.95, 1.29)	(1.00, 1.77)*	(0.90, 1.94)	(0.87, 1.41)		(0.84, 1.29)	(0.91, 1.97)
Model 2, HR	1.23	1.13	Reference	1.07	1.13	1.24	1.05	Reference	1.03	1.28
(95% CI)	(0.91, 1.66)	(0.95, 1.33)		(0.92, 1.25)	(0.84, 1.50)	(0.83, 1.84)	(0.82, 1.35)		(0.83, 1.28)	(0.87, 1.88)
Model 3, HR	1.35	1.15	Reference	1.13	1.33	1.29	1.04	Reference	0.98	1.34
(95% CI)	(0.98, 1.87)	(0.96, 1.38)		(0.95, 1.33)	(0.98, 1.80)	(0.86, 1.93)	(0.80, 1.35)		(0.78, 1.23)	(0.90, 1.98)

Model 1 was adjusted for age and socioeconomic index
Model 2 was stratified by BMI category and adjusted for age, socioeconomic index, marital status, smoking, alcohol intake, physical activity, hypertension, use of lipid-lowering medication, shift work, psychological stress and sleep quality
Model 3 additionally excluded the first 3 years of follow-up

*p<0.05 vs reference

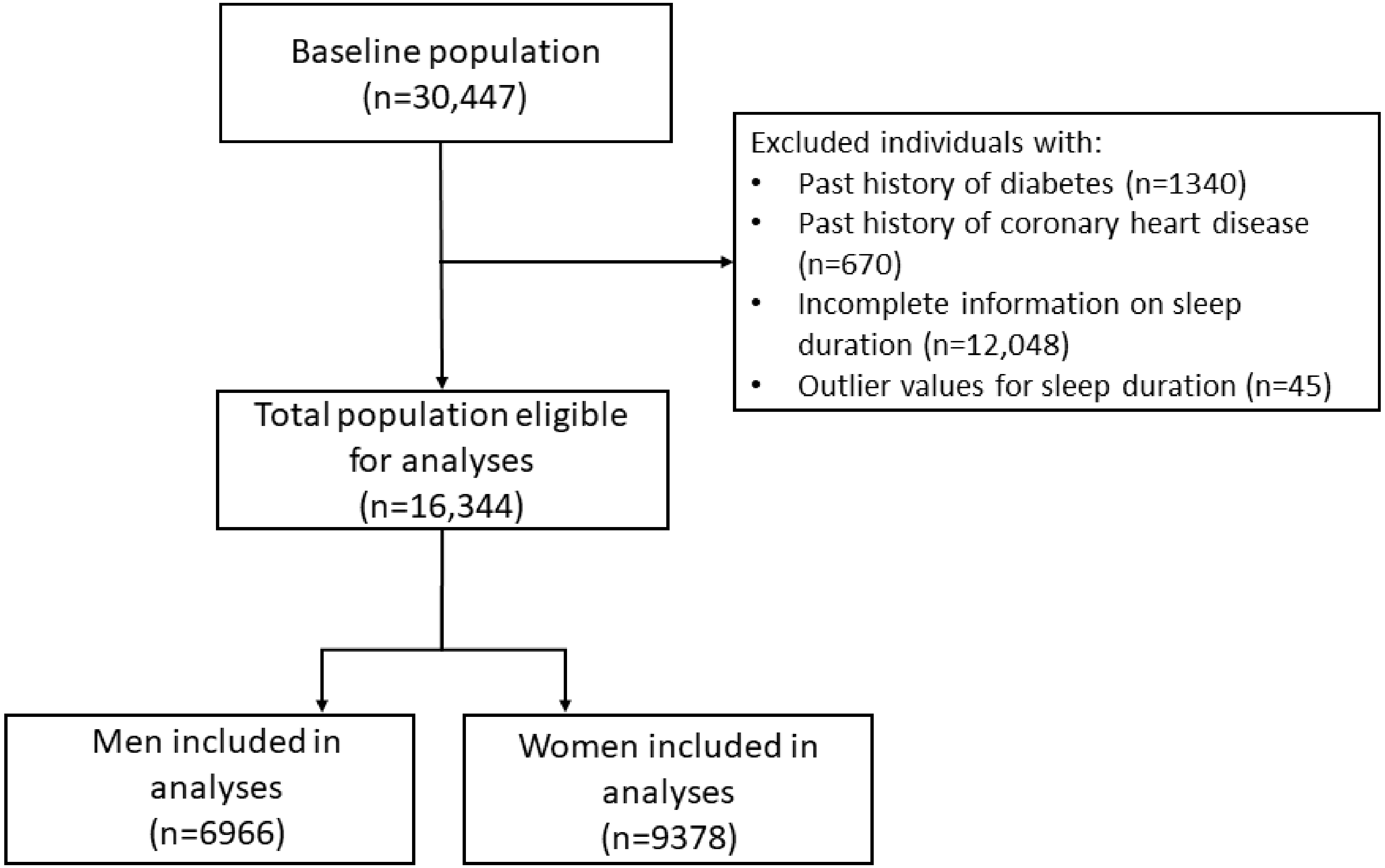

ESM Fig. 1. Flow-chart of the inclusion and exclusion of study participants.
